# Exploring the predictive value of the evoked potentials score in MS within an appropriate patient population: a hint for an early identification of benign MS?

**DOI:** 10.1186/1471-2377-12-80

**Published:** 2012-08-22

**Authors:** Nicolò Margaritella, Laura Mendozzi, Massimo Garegnani, Raffaello Nemni, Elena Colicino, Elisabetta Gilardi, Luigi Pugnetti

**Affiliations:** 1 Laboratory of Clinical Neurophysiology, Scientific Institute (IRCCS) S. Maria Nascente, don C. Gnocchi Foundation, Via Capecelatro 66, Milan, 20148, Italy; 2 Multiple Sclerosis Rehabilitation Unit, Scientific Institute (IRCCS) S. Maria Nascente, don C. Gnocchi Foundation, Via Capecelatro 66, Milan, 20148, Italy; 3 Neurological Rehabilitation Unit, Scientific Institute (IRCCS) S. Maria Nascente, don C. Gnocchi Foundation, Via Capecelatro 66, Milan, 20148, Italy; 4 Department of Agriculture Food and Environmental Economics, University of Milan, Via G. Celoria, Milan, 2-20133, Italy

**Keywords:** Multiple Sclerosis, EP score, Disability prediction, Multivariate analysis, ROC analysis, Benign MS, Evoked potentials

## Abstract

**Background:**

The prognostic value of evoked potentials (EPs) in multiple sclerosis (MS) has not been fully established. The correlations between the Expanded Disability Status Scale (EDSS) at First Neurological Evaluation (FNE) and the duration of the disease, as well as between EDSS and EPs, have influenced the outcome of most previous studies. To overcome this confounding relations, we propose to test the prognostic value of EPs within an appropriate patient population which should be based on patients with low EDSS at FNE and short disease duration.

**Methods:**

We retrospectively selected a sample of 143 early relapsing remitting MS (RRMS) patients with an EDSS < 3.5 from a larger database spanning 20 years. By means of bivariate logistic regressions, the best predictors of worsening were selected among several demographic and clinical variables. The best multivariate logistic model was statistically validated and prospectively applied to 50 patients examined during 2009–2011.

**Results:**

The Evoked Potentials score (EP score) and the Time to EDSS 2.0 (TT2) were the best predictors of worsening in our sample (Odds Ratio 1.10 and 0.82 respectively, p=0.001). Low EP score (below 15–20 points), short TT2 (lower than 3–5 years) and their interaction resulted to be the most useful for the identification of worsening patterns. Moreover, in patients with an EP score at FNE below 6 points and a TT2 greater than 3 years the probability of worsening was 10% after 4–5 years and rapidly decreased thereafter.

**Conclusions:**

In an appropriate population of early RRMS patients, the EP score at FNE is a good predictor of disability at low values as well as in combination with a rapid buildup of disability. Interestingly, an EP score at FNE under the median together with a clinical stability lasting more than 3 years turned out to be a protective pattern. This finding may contribute to an early identification of benign patients, well before the term required to diagnose Benign MS (BMS).

## Background

In the neuroimaging era the role of evoked potentials as diagnostic tools has been greatly diminished. This led many authors to explore how Evoked Potentials (EPs) could still be useful as predictors of clinical disability in multiple sclerosis (MS) [1-11]. Part of the recent literature addressed this problem from a multivariate parametric perspective by looking at the absolute latencies of multimodal EPs [6,7,10,11], whereas in another group of studies individual EPs measures were first transformed into an ordinal summary score (the EP score) [2-5,8,9]. Given that different EP scores as well as different statistical approaches have been proposed, it is perhaps not surprising that a general consensus over both methodology and results is still to be found. A question strictly confined to the choice of the best EP score or the best statistical model is for whom the prediction is most appropriate. Some studies have assessed the predictive value of the EP score by selecting patients with long disease duration and a moderate to severe clinical disability at First Neurological Evaluation (FNE) [2,3,5], while others have enrolled patients with short disease duration and low clinical disability at FNE [2,4]. The definition of the appropriate patient population is decisive for the relation between clinical and subclinical variables, e.g. high values of EP score are more likely to be correlated with high values of EDSS, thus the prediction of disability could be automatically improved in a patient population with a large percentage of secondary progressive MS courses; however, the clinical utility of this choice is questionable because the interest in predicting worsening in patients who are already significantly impaired is low.

Our aim was to evaluate the predictive value of the EP score first by determining how an appropriate patient population should be defined, and then by assessing the performance of the EP score in a multivariate logistic regression analysis. As the EP score summarizes the quantitative information of different EP modalities, it is an optimal tool to evaluate the overall subclinical impairment of MS patients. By analyzing its performance within an appropriate patient population it should be possible to better evaluate the unbiased ability of this score to predict worsening in MS. In particular, the identification of a group with a low risk of worsening may contribute to an early identification of putative benign MS patients [12].

## Methods

### Patients

A total of 143 MS patients, who were referred to our centre (Scientific Institute S. Maria Nascente, don Gnocchi Foundation, Milan, Italy) during the period 1989–2009 for clinical, neuroimaging and neurophysiological assessments, were retrospectively selected from our clinical database. Inclusion criteria for this study were: (1) a diagnosis of Relapsing Remitting MS (RRMS) using Poser [13] and McDonald criteria [14]; (2) at least one complete EPs evaluation performed at FNE (F_EP score) including Visual (VEP 30’ and 15’), auditory (BAER) and somatosensory (SEP lower limbs LL and upper limbs UL) evoked potentials; (3) EDSS at FNE (F_EDSS) lower than 3.5 points assessed by the same trained neurologist using the pre-neurostatus version; (4) at least 2 EDSS follow-ups before the last assessment (5) last EDSS (L_EDSS) and last EPs (L_EP score) assessments for all patients during 2008–2009. Additionally, it has been possible to measure the time between FNE and EDSS 2.0 (TT2) in 65% of the patient population with F_EDSS below 2.0. Thirty-one patients were defined as benign MS according to the last accepted criteria, i.e. EDSS ≤ 2.0 for at least 10 years [12].

At FNE, 10 patients (7%) were receiving immunomodulatory treatments and 16 (11%) immunosuppressive treatment; however, patients with and without treatments did not differ in terms of EDSS, EP score and disease duration at FNE.

Patients with incomplete EP tests or missing EDSS values, as well as patients with EDSS ≥ 3.5 at the FNE were not included. EDSS was considered only if assessed during periods of clinical stability.

This study has been approved by the ethics committee of the Scientific Institute S.Maria Nascente of Milan and has been performed in accordance with the ethical standards of the declaration of Helsinki.

### The EP score

VEP, BAER ,SEP-LL and SEP-UL were recorded according to recommended standardized protocols [15]. SEPs were obtained by electrical stimulation of the median nerves at the wrists, and of the posterior tibial nerves at the ankles. Latencies of the main peripheral, spinal and cortical components were measured along with the latency difference N9-N20 and amplitude difference N20-N25 for median nerve SEP, the latency difference N19-P37 and amplitude difference P37-N45 for tibial nerve SEP. VEP to black and white pattern-reversal stimulation with checks of 30 and 15 minutes of arc were recorded over Oz of the 10–20 international system, with Fz as the reference. The latency of the P100 component and the amplitude difference N75-P100 were measured. BAER to clicks at 70 dB above subjective threshold with contralateral white noise masking were recorded at the CZ electrode referred to the ipsilateral and contralateral ears. The latency of the main peaks I, III and V, the inter-peak latencies (I-III, III-V and I-V) and the I:V amplitude ratio were measured. Stimulation and recording were carried out using commercial biomedical recording systems (Nicolet® CA 2000 and XLTEK® Protektor) by the same experienced technicians. As abnormalities were quantified separately for each modality (VEP 30’, VEP 15’, BAEP, SEP LL, SEP UL), according to a six-point graded scale drawn from the work of Jung et al. (0 = normal; 1 = pathological side difference of latency; 2 = latency above the normal range but below 1.1 x upper limit, or >50% side difference of amplitude; 3 = latency 1.1-1.3 x upper limit; 4 = latency above 1.3 x upper limit; 5 = absent EP component), the worst possible score summarizing all the EPs modalities was 50 (5 points x 2 sides x 5 EP modalities) [4].

### Statistics

First, the associations of the outcome variable L_EDSS with EP score, F_EDSS and TT2, were analyzed by the Spearman’s rank correlation coefficient. In addition, the associations between L_EDSS and other clinical characteristics at FNE were examined. Receiver operating characteristic curves (ROC) for the clinical variables (EP score, EDSS, TT2) were used to predict whether clinical worsening, defined as crossing the threshold of EDSS 3.5, occurred before 2008–2009. The area under the curve (AUC) and the 95% confidence intervals (CI) for each variable were also considered. In line with C. Renoux [16], an EDSS of 3.5 was selected as a turning point between low and moderate/severe disability since it allows a more conservative approach than higher thresholds (e.g. 4.0 or 6.0) by decreasing false negatives.

A change of 1.0 EDSS point is another measure of worsening proposed in literature [5,6]; however, in a clinical sample with random follow-ups a progression of 1.0 or 1.5 EDSS points could be more easily lost than in an experimental sample with follow up visits at fixed intervals. Moreover, this choice would force us to consider changes between different EDSS steps as equal (e.g. 0–1.0 and 2.0-3.0).

Second, we sought to ascertain whether a combination of EP score and other clinical variables could improve the prediction of clinical worsening by using a multivariate logistic regression analysis. To identify other potential predictors of clinical worsening we performed a backward selection starting with a model that contained all demographical (age at FNE, gender, disease duration) and clinical variables (F_EDSS,F_EP score, TT2) with p< 0.2 on bivariate logistic regressions. In the resulting multivariate logistic regression model, variables with p-values ≥ 0.2 were eliminated leaving the F_EP score, F_EDSS and TT2 as the best predictors of disability. Given the multicollinearity between F_EDSS and TT2, which cast doubt on their independence, two models were tested: (model 1) with F_EP score and TT2 and (model 2) with F_EP score and F_EDSS. The final choice of Model 1 (hereinafter referred to as “the model”) was based on likelihood ratio chi-squares, Akaike’s information criteria (AIC) and Bayesian information criteria (BIC). ROC curves were used again to assess the best cut off point of the model in terms of sensitivity and specificity, and the resulting AUC was compared with those of the previous bivariate ROC analyses. The distribution of predicted probabilities for each variable was evaluated by plotting each regressor against the probability of reaching EDSS 3.5. To better assess how final variables might interact, the predicted probabilities were also analyzed by dividing the whole sample by the median value of the variable TT2. The EP scores of the resulting two subgroups (i.e. patients below and above the median of TT2) were plotted against the probability of reaching EDSS 3.5 to assess if the predicted probability curves had a similar shape (i.e. no effect of TT2 subgroups over the EP score) or if differences occurred between subgroups. The same procedure was used after dividing the whole sample by the median value of the EP score.

Third, the model was validated by using a non-parametric bootstrap analysis and it was prospectively applied to a group of 50 patients (11 follow up patients and 39 newly selected cases) examined during 2009–2011 to show its practical utility.

## Results

The demographical and clinical characteristics of the 143 MS patients are shown in Table 1.

**Table 1:** Demographical and clinical variables

**Variable**	**Mean±SD**	**Median (IQR)**
Age at FNE	31.9 ± 8.7	31 (25–38)
Time from first symptom to FNE	4.5 ± 4.6yrs	3 (1–7)yrs
Time from FNE to 2009	10.5 ± 4.6yrs	11(6.5-14)yrs
F_EP score	7.6 ± 8.5	5 (2–11)
F_EDSS (<3.5)	1.3 ±0.9	1.5 (1–2)
TT2	6.5 ± 7.7	3( 0–11)
L_EDSS	2.7 ± 1.7	2.5 (1.5-3.5)

*IQR* = Inter Quartile Range; *FNE* = first neurological evaluation; *F_EP* score= EP score at first neurological evaluation; *F_EDSS* = EDSS at first neurological evaluation; *L_EDSS* = last EDSS assessed during 2009; *TT2*=time to EDSS 2.0; Yrs=years. % women = 79 (n=113).

Spearman correlation coefficients (ρ) between EP score and EDSS ranged from weak (0.27; p<0.001) at FNE to moderate (0.41; p<0.0001) at the time of the last assessment (L_EDSS). Likewise, TT2 was strongly correlated with F_EDSS (−0.73; p<0.0001) and moderately with L_EDSS (−0.55; p<0.0001). The correlation between F_EP score and TT2 was weak (−0.25; p<0.01) and none of the correlations between L_EDSS and the demographical variables were significant. To verify whether the variability of F_EDSS and of the time elapsed from the first symptom to FNE could have influenced the correlation between EP score and disability, we repeated the correlations stratifying cases by the median EDSS value (1.5) and by the interval between disease onset and the first neurophysiologic evaluation [5] (see Table 2 for details).

**Table 2:** Correlations between EP score and EDSS

**Variable**	**At FNE**		**At last follow-up**	
	**ρ**	***p*-value**	**ρ**	***p*-value**
(**a**)F_EDSS				
≤ 1.5(n=94,EP score 6.4±7.5, Disease duration 3.7±4.1)	0.14	0.15	0.33	0.002
>1.5(n=49, EP score 10±9.8, Disease duration 5.9±5.1)	0.39	0.0114	0.41	0.0064
(**b**)Interval between MS onset and FNE				
< 2 yrs (n=48)	0.08	0.55	0.46	0.0027
2-6 yrs (n=55)	0.20	0.13	0.34	0.0297
> 6 yrs (n=40)	0.47	0.006	0.46	0.009

(a) patient population divided for the median EDSS; (b) patient population stratified for the interval between onset and the first neurological evaluation (FNE); p-values are corrected for multiple comparisons; *F_EDSS*= first EDSS assessment.

We found a statistically significant correlation between the L_EP score and L_EDSS in both subgroups, while at FNE only the subgroup with an EDSS > 1.5 was significantly correlated with F_EP score (Table 2). Forty-eight patients had an EPs test within 2 years from disease onset, 55 after 2–6 years, and 40 after more than 6 years. We found that the correlation between the L_EP score and L_EDSS was significant in all three subgroups, while at FNE the correlation between the F_EP score and F_EDSS was significant only for the longest duration subgroup (Table 2).

The predictive power of the F_EP score, F_EDSS and TT2 was analyzed by means of ROC curves: AUC for the F_EP score was 0.72 (95 % CI: 0.63-0.82), 0.71 for F_EDSS (95% CI: 0.61-0.81), and 0.74 for TT2 (95% CI: 0.66-0.82).

Our backward selection procedure for the multivariate logistic regression resulted in the following prediction model (Table 3):

$$\log P\left(Y=1|x_{1}\ldots x_{n}\right)=-0.899+0.095*\left[EPscore\right]-0.199*\left[TT2\right]$$

where *n*=2 and $LR\chi^{2}=45.84\left(p>\chi^{2}=0.0000\right)$

**Table 3:** The logistic regression model

**Parameter**	**β-Estimate**	**Odds ratio**	**Standard Error**	***p*-value**
*Model 1:*				
Intercept	−0.8987	-	0.3500	0.010
EP score	0.0955	1.10	0.0282	0.001
TT2	−0.1990	0.82	0.0587	0.001

All the diagnostics of the logistic regression model were run to check for possible errors of specification, multicollinearity and influential observations. Predictions showed a moderate correlation with the observed values (Spearman rank ρ = 0.495, p<0.0001). The model was tested against the corresponding bivariate models including either TT2 or F_EP score to evaluate how the addition of each variable contributed to the model fitting. The goodness of fit was significantly higher in both cases (likelihood ratio χ² = 22.69, p<0.0001 for the inclusion of TT2 and likelihood ratio χ² = 14.49, p=0.0001 for the inclusion of EP score); indeed the AUC was 0.8135 (95% CI 0.74 – 0.88), better than the AUCs of the EP score, F_EDSS and TT2 taken individually (p <0.02).

**Figure 1:**
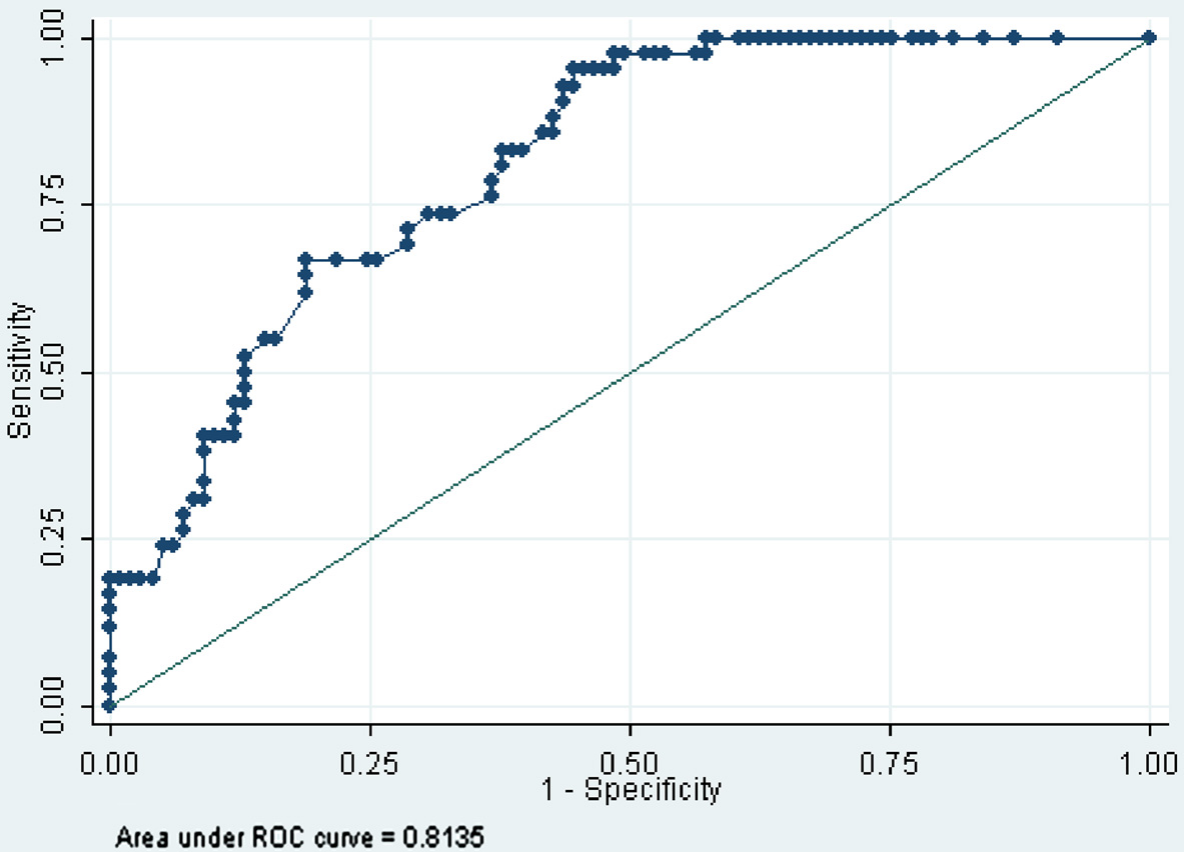
**ROC curve resulting from the logistic regression model.** The area under the curve (AUC = 0.81) shows the sensitivity and specificity corresponding to different cut-off points of the prediction of clinical worsening (defined by patients reaching the threshold of EDSS 3.5). The best cut-off point defined by the maximum of Youden’s index corresponds to a sensitivity of 0.738 and a specificity of 0.693.

The sensitivity and specificity at different cut-off points for the prediction of clinical worsening are shown in Figure 1.

The cut-point for the predicted threshold of EDSS 3.5 was 0.31, corresponding to the highest sensitivity (0.738) and the highest specificity (0.693). The resulting index of accuracy (Youden) was 0.43.

**Figure 2:**
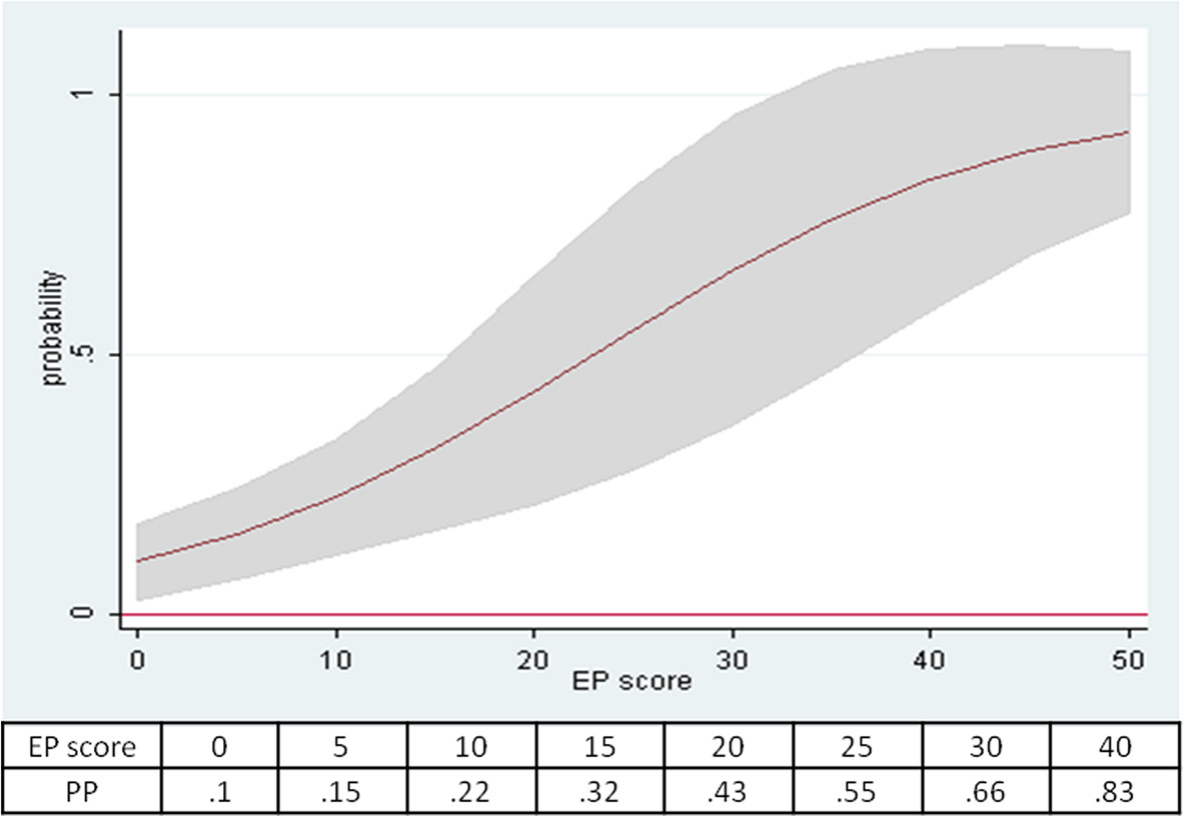
**Distribution of predicted probabilities for EP score and TT2.** A sampling plot drawn from the logistic regression model showing how the predicted probabilities (PP) of clinical worsening are distributed along the EP score scale (with TT2 held constant at the mean). A sample of EP values and their corresponding PP is reported below the graph. Shaded area represents 95% CI.

**Figure 3:**
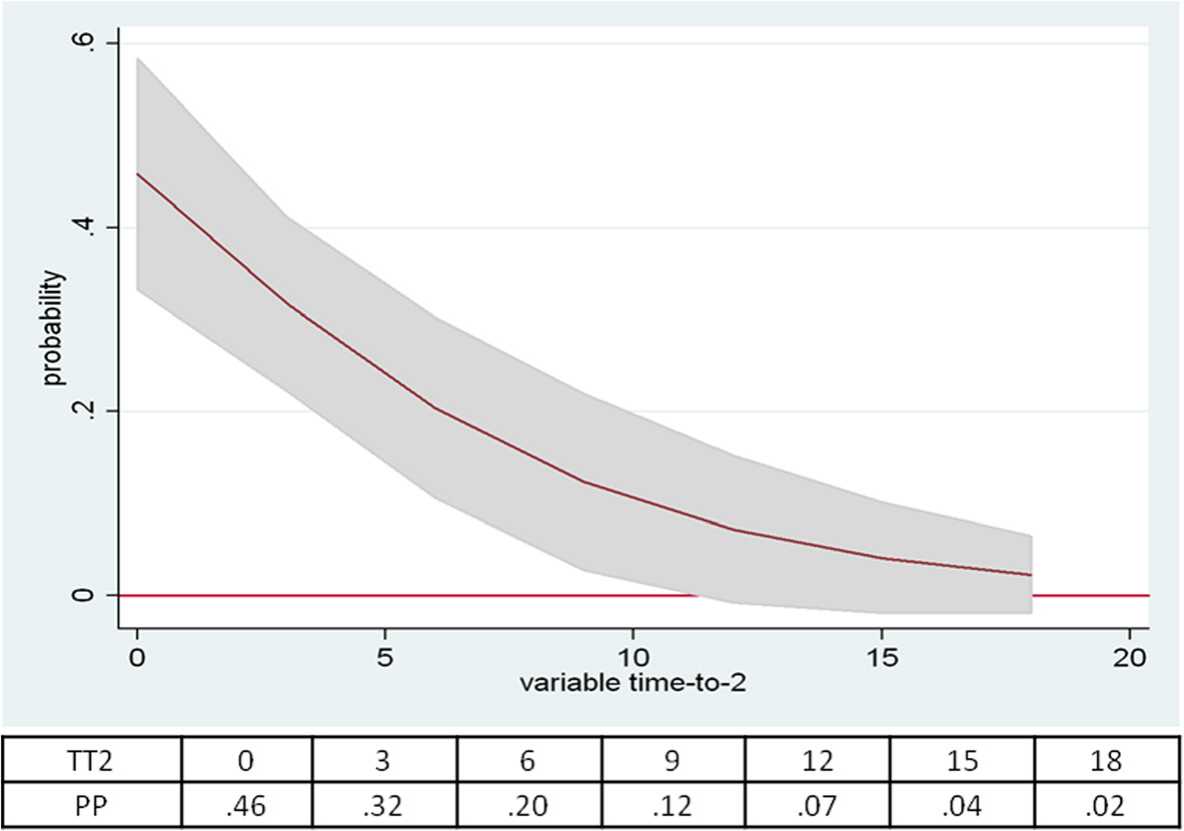
**Distribution of predicted probabilities (PP) for EP score and TT2.** A sampling plot drawn from the logistic regression model, showing how the predicted probabilities of clinical worsening are distributed along the TT2 variable (x-axis = yrs; with the EP score held constant at the mean). A sample of TT2 values and their corresponding PP is reported below the graph. Shaded area represents 95% CI.

The predicted probabilities of the model are plotted in Figure 2 and 3; it appears that the probability to reach EDSS 3.5 increases as the F_EP score approaches the highest values of the scale; over 25 points, worsening is highly probable (Figure 2). On the other hand, the probability decreases as the length of time (years) to reach EDSS 2.0 increases; over 12 years, worsening is highly improbable (Figure 3).

**Figure 4:**
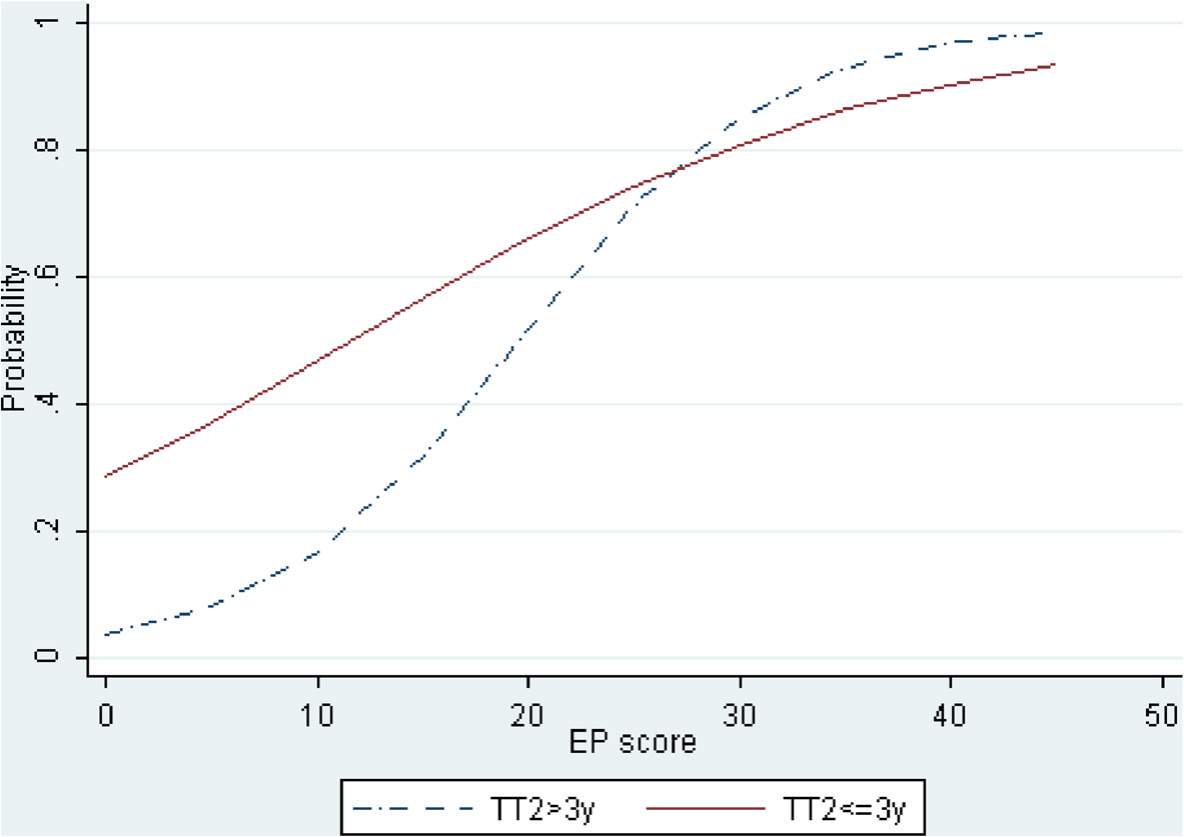
**Plot of predicted probabilities (Y-axis) vs. the EP score (X-axis).** The dotted line represents patients with TT2 > 3 yrs; the solid line represents patients with TT2 ≤ 3 yrs. The difference between the curves is largest at the origin of the EP axis and tends to decrease as the EP values grow until it eventually becomes negligible at approximately an EP score of 20.

**Figure 5:**
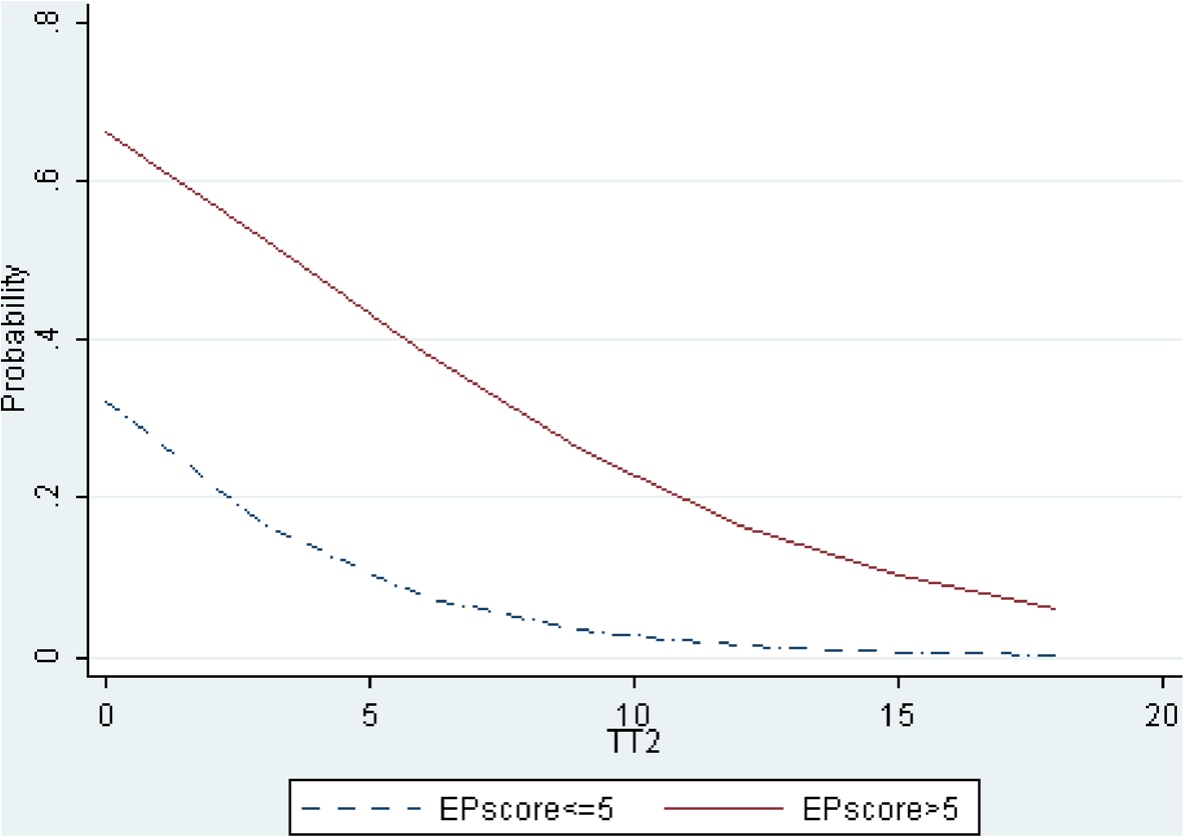
**Plot of predicted probabilities (Y-axis) vs. TT2 (X-axis).** The dotted line represents patients with an EP score ≤ 5; the solid line represents patients with an EP score > 5. The difference between the curves is largest at the origin of the TT2 axis and decreases as TT2 exceeds 4–5 years.

A more detailed picture was obtained by dividing the whole sample by the median value of each variable (3 years for TT2 and 5 points for EP score), as detailed in the Methods. Accordingly, Figure 4 shows that the TT2 subgroups (i.e. above and below the median of TT2) show diverging probability curves below 20–15 points, whereas at higher EP values the curves tend to overlap. Likewise, the EP score subgroups (i.e. above and below the median of EP score) show diverging probability curves associated with a length of time to EDSS 2.0 shorter than 3–5 years, while at longer TT2 values the curves tend to overlap (Figure 5).

It is worth clarifying at this point that these results were obtained by arbitrarily ending the study in 2009. This means that theoretically the prediction could be extended up to our longest retrospective analysis (20 years, from 1989 to 2009); however, as the time from FNE to the end of the study was greatly variable among our patients, the actual prediction span is lower than 20 years (10 years on average, see Table 1). In order to overcome this limitation, data from 11 patients (7.7% of the original sample), who were followed up for less than 5 years between FNE and the study endpoint, were pooled with data of 39 new patients examined during 2009–2011 and used for a prospective assessment of the model (see Table 4 for details).

**Table 4:** Results of the prospective study

**prospective study**	**F_EP score (med; range)**	**F_EDSS (med; range)**	**Pr.<0.4(n/ms)**	**Pr.0.4-0.6 (n/ms)**	**Pr.≥0.6(n/ms)**
*11 pts.*	5; 0-25	1.5; 0-3	6/0	3/1	2/0
*6 pts.*	1.5; 0-37	2.0; 0-3	4/0	-	2/0
*33 pts.*	6; 0-28	1.5; 0-3	26/.	4/.	3/.

11 pts. = 11 patients drawn from the original patient population who returned for additional EDSS assessment during 2010–2011; 6 pts. = 6 patients with 2 examinations during 2009–2011; 33 pts.= 33 patients with only 1 EPs and EDSS assessment during 2009–20011. *Med* = median; *Pr*. = probability of worsening; *n/ms* = number of patients/ number of patients misclassified by the model; *F_EP score* = EP score at first neurological evaluation; *F_EDSS*= EDSS at first neurological evaluation.

Ten of the 11 patients who were reassessed once during the prospective follow-up were correctly classified by the model. The only misclassified case had an F_EP score (dating back to 1996) of 7 and a F_EDSS of 2.0 resulting in a probability of 0.44, which lies just below the model cut off and indicates a low probability of progression toward EDSS 3.5. Despite this prediction was still correct in 2009, this patient eventually progressed to EDSS 3.5 during 2011, i.e. 15 years after FNE.

All the patients with a probability exceeding 0.6 (n= 4/17) having a mean F_EP score of 25.7 and an F_EDSS over 2.0 reached the threshold in a variable time span ranging from 1 to 20 years. On the other hand, patients with a probability lower than 0.4 (n= 10/17) and a mean F_EP score of 1.4 did not reach the threshold within a 16 years period; five of them had not reached EDSS 2.0 at the time of L_EDSS assessment, while the others had a mean TT2 of 4.2 years.

Finally, a non parametric bootstrap analysis was carried out to validate the model using the Bias Corrected and Accelerated (BCA) method in order to estimate the 95% bootstrap confidence interval for each variable (EP score CI= 0.04 to 0.15 ; TT2 CI= −0.13 to −0.29). Given that the diagnostic criteria for bootstrap analysis were fully met (low differences between predicted and observed regressors coefficients and standard errors, Gaussian shape of the bootstrap distribution), we interpreted these results as consistent with a successful prospective validation of the model.

## Discussion

The retrospective part of this study aimed to build up and evaluate a model combining neurophysiologic and clinical evaluations to obtain a reliable prediction of the progression of disability in MS patients with particular attention to the role of evoked potentials. A summary score considering both abnormalities of latencies as well as of morphology and of amplitude symmetry of the principal EP components [4] was utilized as input in the analyses to assess the prognostic value of EPs. Since none of the recent works have compared the different EP score systems which have been proposed in the last decade, consistent with a previous work of our group [8], we chose the scoring system preserving most of the EP information [4]. The latter allows a maximum of 6 points for each side and for any of 5 EP modalities, as opposed to only 4 points in Leocani et al. [3] and 3 in Kallman et al. [2].

**Figure 6:**
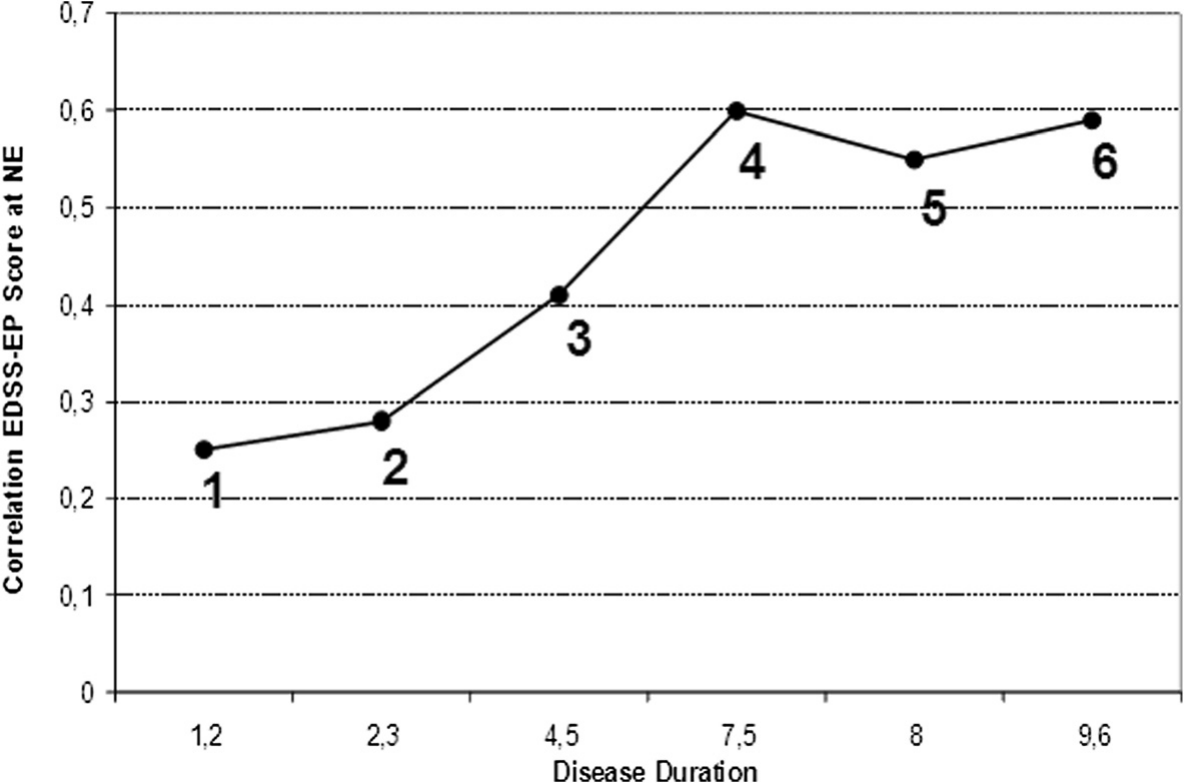
**Correlations between EDSS and EP scores in the last 6 years literature.** The correlations between EDSS and EP scores reflect the researchers’ choice of patients selection criteria. **1**: Kallmann et al. 2006 [2] group 1, F_EDSS =2.0, range (0–4). **2**: Jung et al. 2008 [4] F_EDSS = 1.5, range(0–3). **3**: this study, F_EDSS = 1.5, range(0–3). **4**: Leocani et al. 2006 [3], F_EDSS = 3.5, range (1–8). **5:** Invernizzi et al. 2011 [5], F_EDSS = 3.0, range(0–6.5). **6**: Kallmann et al. 2006 [2] group 2, F_EDSS =3.5, range(0–7).F_EDSS= first EDSS assessment.

At FNE, our patients showed a correlation between EP score and EDSS which was lower compared to that reported by Invernizzi et al., Leocani et al., and Kallman et al.’s group 2 [2,3,5], but greater than that reported by Jung et al. and Kallman et al.’s group 1 [2,4]. As shown in Figure 6, this apparent inconsistency is likely due to two important factors. First, the correlation between EDSS and EP score depends on the severity of disability already present at FNE insofar as the correlation tends to increase as disability builds up (Figure 6, from 1 to 6). As already suggested by Leocani et al. [3], this is a pattern toward a ceiling effect that results from the inclusion of subjects with more severe disability and a progressive disease course.

Second, disease duration also impacts the degree of clinical disability and, consequently, the correlation between clinical and subclinical measures. This is clearly shown in Figure 6 (x-axis) where the correlation between EDSS and EP score tends to grow as the disease duration increases because so does the F_EDSS. The effect of the disease duration showed up more clearly when we analyzed the correlation between F_EP score and F_EDSS after dividing our sample by the median value of F_EDSS (Table 2). The correlation was statistically significant only in the higher F_EDSS subgroup. In contrast to Invernizzi et al. [5], this finding suggests that the difference in disease duration between the 2 subgroups thus identified (3.7 yrs, 5.9 yrs; p=0.005) was decisive. Moreover, when we stratified by the time from the first symptom to FNE, only patients assessed more than 6 years after disease onset showed a moderate correlation (ρ=0.47) between the F_EP score and F_EDSS, while at the last follow-up the correlations increased approximately to the same extent also in the two remaining subgroups. These findings are in line with Kallmann et al. [2] who found a significant correlation between F_EDSS and F_EP score in patients with a long disease duration at FNE (mean 9.6 years), while in the group with a shorter disease duration (mean 1.2 years) the correlation was not significant.

Moreover, Hughes et al. [17] recently confirmed that the prediction at 5 and 10 years based on the EDSS is higher when applied to patients with 4–5 years of disease duration. Findings from other fields of research such as MRI are also affected by similar choices: in Onu et al.’s work [18], the authors admitted that significant correlations between MRI and EDSS depended on the inclusion of patients with high EDSS (0–5.5) and a long disease duration (mean = 9.3 years), whereas in a work by Metwalli et al. [19], who studied patients with lower EDSS (0–3) and shorter disease durations (mean = 1.2 years), no significant correlations were found. Though it is certainly true that sampling the entire range of the EDSS can lead to better coefficients whatever the correlate [20], the opportunity of such a choice is questionable as the most critical medical decisions are those made in the early phases of MS.

An early MS diagnosis, in addition to being preferred by MS patients [21], generally also imply low F_EDSS scores; indeed, as recently reviewed by C. Renoux [16], a shorter time from onset to moderate disability (DSS 3.0 or 4.0) has been associated with a faster rate to severe disability; moreover, worsening in MS becomes more common after EDSS 4.0 [17], further remarking the importance to include only patients with low F_EDSS if useful clinical predictions are to be made. Accordingly, we could confirm that the variable TT2 represents the early progression of the disease and can predict further worsening. Although it was set at the value of zero for a part of our patient population (35% already with EDSS 2.0 at FNE), the variable TT2 was significant both in bivariate and multivariate analyses and also a significant correlation with L_EDSS (−0.55; p < 0.0001) was rather stable. It is worth reminding here that a threshold of EDSS 2.0 was also recently applied to the definition of BMS (Benign Multiple Sclerosis) together with a disease duration equal or longer than 10 years [12].

Consequently, a logistic regression model including the F_EP score as well as the TT2 variable was applied to a sample of 143 RRMS patients having a mean disease duration of 4.5 years and a mean F_EDSS of 1.3. The aim of the model was to predict the progression of disability defined as the risk of reaching the threshold of EDSS 3.5 [16,17]. Likelihood ratio tests successfully assessed the relevance of the overall model (likelihood ratio χ² = 45.84, p<0.0001) and of TT2 and EP score variables (likelihood ratio χ² = 22.69, p<0.0001 and likelihood ratio χ² = 14.49, p=0.0001 respectively). The multivariate approach was supported also by a significant improvement of the AUC compared with that previously obtained with single variables (AUC: 0.8135, p<0.02). A first consideration is that the EP score has a strong prognostic value when EDSS 2.0 is reached in 3–5 years from FNE (Figure 5), since the difference in the probabilities of further worsening between the two subgroups (i.e. above or below the median value of the EP score) is nearly 30%. This means that when TT2 is short, the probability of worsening switches from 30% to over 60% by having , for example, 4 or 8 points of the EP score respectively.

On the other hand, high EP scores (over 20–25 points) or a long time to reach EDSS 2.0 (over 10–15 years) were not associated with very different probabilities of worsening among the subgroups obtained by dividing the whole sample by the median value of TT2 (Figure 4) or of the EP score (Figure 5). First, this indicates that data of patients with severe subclinical damage (i.e. high EP scores) and patients with progressive MS courses should not be considered for predictive models if the aim is an early identification of different patterns of progression; indeed, patients with these characteristics are well known to be candidates to clinical worsening and no prediction is needed. Second, patients with long disease duration at FNE have to be excluded if long disease duration is not associated to clinical stability. We have shown (Figure 6) that since mean disease duration is related to mean EDSS, worsening is to be expected; by the same token, if a long disease duration is associated to clinical stability, like in BMS, further worsening becomes unlikely and again no prediction is really needed.

The EP score and TT2 have the greatest utility when their values are able to show different patterns of worsening. By dividing our sample in 4 groups, namely (a) high F_EP score + short TT2 (b) high F_EP score + long TT2; (c) low F_EP score + short TT2 and (d) low F_EP score + long TT2, we showed that our model can identify separate patterns. Groups (a) and (b) have in common a high subclinical impairment and therefore are candidates to clinical worsening whatever the conversion time to clinical disability (as shown by overlapping solid and dotted lines approximately in the last 30 values of the x-axis in Figure 4). On the other hand, in the case of groups (c) and (d) characterized by a low EP score, the difference in TT2 determines a higher probability of worsening to group (c) than to group (d) (solid and dotted lines in the first 5 values of the x-axis in Figure 5). A high subclinical impairment (i.e. high EP score) is likely to reflect a massive attack of MS to the central nervous system and the probability that the adaptive brain responses will not be sufficient to compensate the damages; thus the disability will likely worsen whatever its speed of progression. On the other hand, a low subclinical impairment could enable compensation processes. In this case, even small differences in EP score and speed of disability progression (TT2) may identify patients in whom compensatory processes can be successfully enabled, like in benign MS [21,22]. Accordingly, Figure 5 shows that as the time from FNE to EDSS 2.0 increases, the chance of further worsening decreases following two probability curves depending on the F_EP score; when the EP score is above the median value, the probability takes about 15 years without clinical progression to become negligible (<10%). On the other hand, when the EP score is below the median value, the probability of further worsening approaches 0 after only 4–5 years without clinical progression. This last finding gives the EPs a possible role in the debate concerning the definition of BMS [23-25]. According to an earlier EDSS-based definition of BMS a patient with a final EDSS ≤ 3.0 was declared as “benign” after 15 years from onset [26]. Recently, the diagnostic criteria for BMS have been redefined as an EDSS score ≤ 2.0 after a disease duration of at least 10 years [12]. However, the debate has been reopened by recent reports which state that cognitive impairment was detected in 45% of a large group of patients fulfilling traditional criteria for BMS [27], but also by the suggestion that neuropsychological tests can contribute to a more accurate identification of “true” BMS [23,27-29]. In this study we provide evidence that the EP score may be an interesting covariate for the definition of BMS. Indeed, the risk of worsening is almost null in patients with a F_EP score lower than 5 points and a time to EDSS 2.0 of 4 or more years (dotted curve in Figure 5, representing 22% of the patient population). As shown in Figure 5, our results are in line with the earlier and last stringent definition of BMS because the probability goes under about 10% after 10 years and under 5% after 15 years; furthermore, the dotted curve indicates that for patients with low F_EP score, the risk becomes less than 10% after about 4–5 years if the EDSS remains below 2.0, and already tends to zero between 5 and 10 years. This finding suggests that the information drawn from the EPs could substantially improve the sensitivity and decrease the time needed to make a diagnosis of BMS according with the last criteria [12]. We acknowledge however, that the failure to include the motor evoked potentials - not available for the entire patient population and which have been shown to be significantly correlated to the EDSS score [2-7,9-11] - and other potentially useful covariates such as neuropsychological tests [27-29] could have reduced the accuracy of our prediction. It is our belief that a protocol combining all the variables of interest for the prediction of disability should be encouraged.

As recently underlined by Schlaeger et al. [6,7], predictions based on EPs do not seem to be influenced by immunomodulatory treatments. Vucic S [31] suggested that this fact may imply an element of disease irreversibility already at the time of initial assessment [31]. If it was true, it would be advisable to reconsider the therapeutic and monitoring approach to BMS in the light of early predictions based on appropriate multivariate models. This also appears to support the need for further studies employing sensory and motor EPs together with neuropsychological tests to provide a more reliable prediction of BMS.

In the prospective part of this work we evaluated the risk of progression to EDSS 3.5 by applying our model to data partially obtained during the period 2009–2011. The outcome was correctly predicted by the model in 16 of 17 patients who completed the two years follow-up; the subject who was misclassified received a prediction close to 0.5. To improve the usefulness of the model and reduce false negatives, we are paying special attention to patients with a predicted probability in the range between 0.4 and 0.6. Four of the 33 patients who were assessed only once during 2009–2011 fulfilled this requirement and are now being closely monitored.

## Conclusions

In conclusion, we showed that a logistic regression model combining clinical and neurophysiologic data collected at FNE from early RRMS patients can be a reliable tool to identify patterns of prognosis in everyday clinical practice. Furthermore, we have been able to identify a pattern that could improve the definition of BMS using both EP score and EDSS progression. We have also discussed why a model centered on an appropriate patient population, i.e. RRMS with low disease duration and low F_EDSS, is to be preferred to models derived from samples with higher disease duration, higher F_EDSS and progressive MS courses which lead to more accurate but less practical predictions. We strongly believe that heuristic rather than esthetic results are to be pursued and that the real challenge arena for prediction models in MS is the early phase of the disease when divergence among clinical and neurophysiologic measures is still important, allowing the EP score to express its unbiased potentiality.

## Abbreviations

EPs: Evoked potentials; MS: Multiple sclerosis; EDSS: Expanded disability status scale; FNE: First neurological evaluation; TT2: Time to EDSS 2.0; BMS: Benign multiple sclerosis; EP score: Evoked potentials score; F_EP score: EP score at FNE; L_EP score: Last EP score assessment; RRMS: Relapsing remitting multiple sclerosis; F_EDSS: EDSS at FNE; L_EDSS: Last EDSS assessment; VEP: Visual evoked potentials; BAER: Brainstem auditory evoked potentials; SEP: Somatosensory evoked potentials; UL: Upper limbs; LL: Lower limbs; CI: Confidence intervals; ROC: Receiver operating characteristic; AUC: Area under the ROC curve; AIC: Akaike's information criteria; BIC: Bayesian information criteria; BCA: Bias corrected and accelerated method; SE: Standard error; MRI: Magnetic resonance imaging; DSS: Disability status scale.

## Competing interests

The authors declare that they have no conflict of interest.

## Authors' contributions

NM conceived and performed computational analysis, statistical analysis, results analysis and participated in manuscript discussion, manuscript preparation, manuscript writing and manuscript review; LM carried out patients recruitment and clinical assessments, participated in results analysis and discussion, helped in manuscript preparation and manuscript review; MG participated in database preparation and analysis, and performed a review of neurophysiologic tests; EG carried out patient collection and performed neurophysiologic tests; EC helped in statistical analysis and manuscript review; RN participated in results analysis, discussion and manuscript review; LP conceived the study, participated in its design, coordinated neurophysiologic analysis, performed results analysis and discussion, helped in manuscript preparation and manuscript review. All authors read and approved the final manuscript.

## Pre-publication history

The pre-publication history for this paper can be accessed here:

http://www.biomedcentral.com/1471-2377/12/80/prepub
